# Adrenomedullin delivery in microsphere-scaffold composite for remodeling of the alveolar bone following tooth extraction: an experimental study in the rat

**DOI:** 10.1186/1475-925X-12-99

**Published:** 2013-10-08

**Authors:** Lin Wang, Ling Zheng, Chunyan Li, Shujun Dong, Lan A, Yanmin Zhou

**Affiliations:** 1 VIP integrated department, Stomatological hospital, Jilin University, Changchun, China; 2 Implant center, Stomatological hospital, Jilin University, 1500# Qinghua Road, Chaoyang District, Changchun, China

**Keywords:** Poly (lactic-co-glycolic) acid, Microsphere, Adrenomedullin, Alveolar bone loss, Bone regeneration

## Abstract

**Background:**

Alveolar ridge resorption, as a significant problem in implant and restorative dentistry, has long been considered as an inevitable outcome following tooth extraction. Recently, adrenomedullin (ADM) is reported to be able to stimulate the proliferation and migration of various cells including osteoblasts. The purpose of this study was to investigate the influence of local ADM application in the tooth extraction socket *in vivo*.

**Methods:**

Chitosan micropheres were developed by an emulsion-ionic cross-linking method for ADM delivery. Poly (L -lactic-co-glycolic) acid (PLGA) and nano-hydroxyapatite (nHA) were used to prepare scaffolds to contain the micrspheres with ADM. *In vivo* experiment was evaluated by transplanting the composite into the rat socket right after the incisor extraction. After 4, 8, 12 weeks implantation, radiographic and histological tests were carried out to evaluate the effect of released ADM on the alveolar bone.

**Results:**

The microspheres had a spherical structure and a relative rough and uniform surface, and the particle size was under a normal distribution, with the average diameter of 38.59 μm. The scaffolds had open and interconnected pores. In addition, the high porosity of the composite was 88.93%. Radiographic and histological examination revealed that the PLGA/nHA/CMs/ADM composite could accelerate the alveolar bone remodeling and reduce the residual ridge resorption compared with the PLGA/nHA/CMs scaffold.

**Conclusions:**

The results of this study suggest that local application of ADM has the potential to preserve the residual alveolar ridge and accelerate the alveolar bone remodeling.

## Background

Nowadays, the dental implant is accepted as a promising approach to replace a single tooth or multiple adjacent missing teeth, or to support a removable prosthesis. The classic theory holds a veiw that the interval time between tooth extraction and implant placement is three months, when the bone condition is insufficient for immediate implant. During the three-month healing time, the resorption of alveolar bone will take place following tooth extraction, which is termed residual ridge resorption. Residual ridge resorption is a common and incapacitating problem, particularly for persons with edentulous aesthetic areas and posterior mandibles. It often leads to a lack of sufficient support for implant placement. The quest for socket preservation is one of the major focuses in order to achieve optimum esthetics and function with implant restorations. Many approaches [1-3] including autograft and allograft have been attempted. However, secondary lesions, short in supply and poor long-term results of these treatments limit their clinical applications [4].

Bone tissue engineering for some time has been considered as a promising alternative approach to autologous bone graft. Artificial bone scaffold for clinic is designed to mimic native extracellular matrix as closely as possible in term of structure and function [5]. Poly (lactic-co-glycolic) acid, short for PLGA, was one of the most widely used biodegradable polymers for scaffold because of its good biocompatibility and controllable degradation rate. However, PLGA is hydrophobic and lack of a cell-recognizing signal surface. The combination of PLGA and a natural polymer, such as hydroxyapatite might overcome some of the limitations [6]. Chitosan has been widely used for the controlled delivery of peptides or proteins in the format of microspheres, due to its excellent biological properties such as biodegradability, biocompatibility, non-toxicity, bacteriostatic and strong adhesion [7].

Recently, the strategies for bone tissue engineering have turned to the application of cytokines and specific growth factors, such as BMP2, VEGF, for accelerating new bone formation *in vivo* via transmitting signal to modulate cellular activities [8]. To our knowledge, not only osteogenesis, but also angiogenesis is a significant property for artificial bone scaffold. Therefore, multifunctional growth factors are eargly required. Adrenomedullin (ADM), as a 52-amino-acid regulatory peptide, was initially isolated from human pheochromocytoma [9]. It is a member of the CGRP (calcitonin gene-related peptide) family because of their structural homology [10]. As reported [11], ADM is an additional potent mitogen to osteoblasts, stimulating the proliferation of primary osteoblasts and osteoblast-like cell lines. Moreover, other significant effects had also been investigated in recent years, such as angiogenesis [12], and antibacterial effects [13]. CORNISH *et al.*[14] suggested ADM could stimulate osteoblast proliferation and increase intracellular Ca^2+^ levels. Further study has inferred that ADM-induced proliferation is due to an increase in [Ca^2+^]_i_ by facilitation of voltage-dependent Ca^2+^ channels [15]. Furthermore, RIBATTI *et al.* demonstrated that ADM possessed a significant proangiogenic effect [16]. Evidence is accumulating that ADM would be a promising therapeutic agent for bone formation.

In the present study, PLGA/nHA and chitosan were chosen as carriers for ADM delivery to overcome the disadvantage of rapid clearance associated with the use of ADM. Firstly, chitosan microspheres (CMs) loaded with ADM were prepared by an emulsion-ionic cross-linking method. Afterwards, CMs with ADM was embedded into PLGA/HA scaffolds by thermally induced phase separation. The purpose of present study was to investigate the effects of local application of ADM in a rat model on alveolar bone rebuilding following incisor extraction, and the efficiency of microspheres-scaffold complex as a carrier for this peptide.

## Methods

### Materials

Chitosan (Mw = 500 kDa) was purchased from Jinqiao Chemical Reagents Company (Taizhou, Zhejiang, China). Human adrenomedullin (purity=95% by HPLC) was obtained from Phoenix Pharmaceuticals (Burlingame, Canada). TPP and span-80 was got from Aladding (Shanghai, China). PLGA (nLA/nGA=80/20) and nHA were gifts from Changchun institute of applied chemistry Chinese academy of sciences. Other chemicals were all analytical grades and used as received.

### Preparation and characterization of scaffolds

Chitosan microspheres were fabricated by an emulsion-ionic cross-linking method described previously [17]. Briefly, 900 mg of chitosan was dissolved in 29 mL of 2% (v/v) aqueous acetic acid, stirring overnight at room temperature. Afterwards, 1 mg of ADM was dissolved in 1 mL of 2% (v/v) acetic acid and added to the chitosan solution. The mixture was poured into 300 mL of liquid paraffin containing 2% (w/v) of span-80 and stirred mechanically for 2 h. Then, 70 mL of 5% (w/v) TPP was slowly added in droplet into the emulsion and stirred for 4 h at room temperature. The microspheres were obtained by washing with excess amounts of petroleum ether, isopropyl alcohol and distilled water repeatedly, prior to lyophilization.

**Figure 1:**
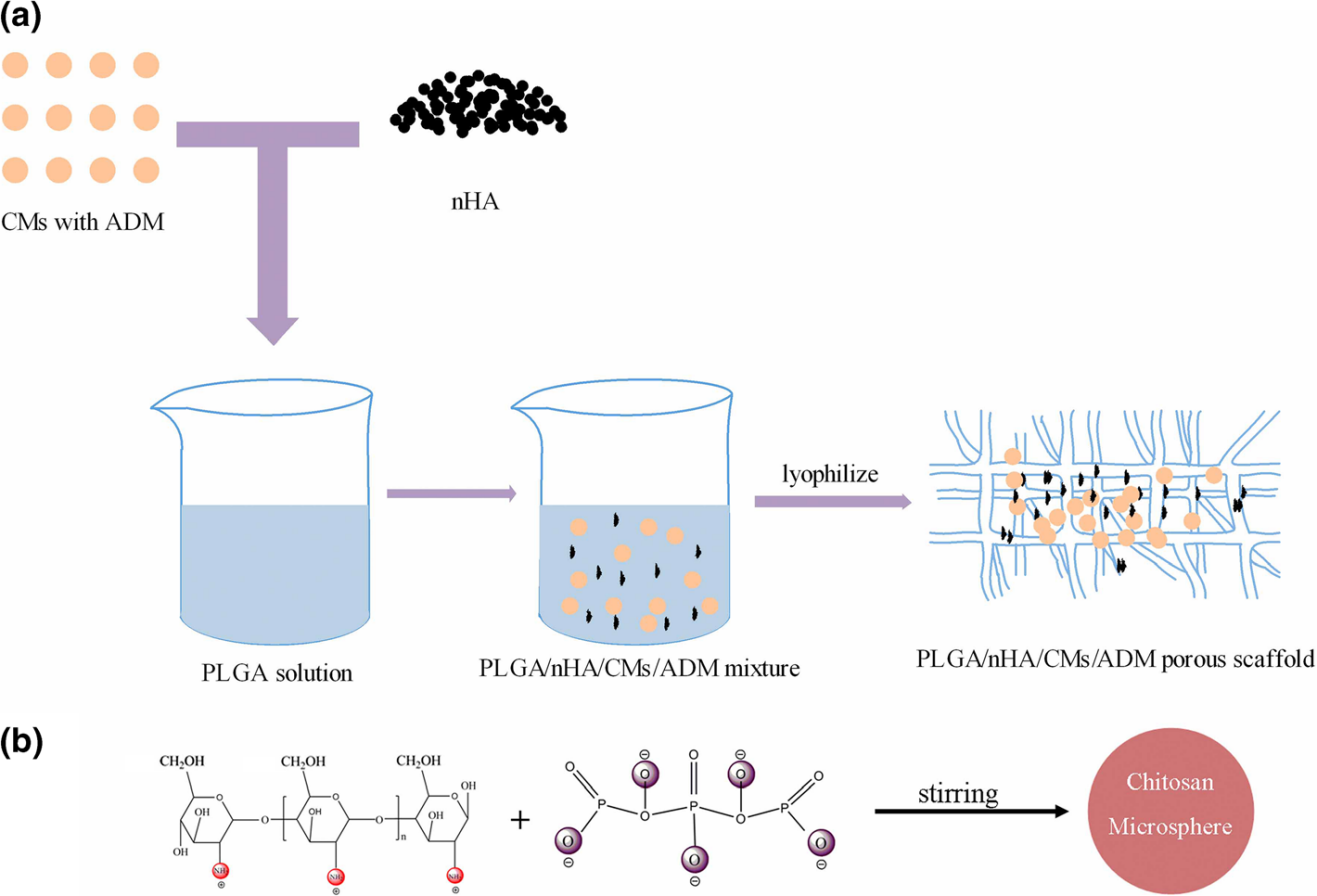
**Schematic illustrations of TPP-chitosan microspheres preparation (a) and microsphere-scaffold composite fabrication (b).** The chitosan microspheres loaded with ADM were prepared by the emulsion method in the presence of TPP, carrying five negative charges which allows the electrostatic interaction with positively charged amino group of chitosan in an aqueous acidic solution.

Microsphere-scaffold composite was developed by thermally induced phase separation outlined in Figure 1a. Firstly, 720 mg of PLGA was dissolved in 12 mL of in 1, 4-dioxane. 360 mg of nHA was added into the mixture after stirring for half an hour. Ultrasonics was used to disperse the nHA completely for 10 min. 240 mg of chitosan microspheres with ADM was added to the above solution subsequently. The mixture was agitated by magnetic stirring to disperse the microspheres completely and poured into a polytetrafluoroethene plate. Afterwards, the solution was frozen in refrigerator at −20°C overnight. Finally, PLGA/nHA/CMs/ADM scaffolds were obtained after lyophilized. The scaffolds without ADM were also prepared in the same manner as control.

The structure of polymer scaffold, as well as chitosan microsphere, was examined by scanning electron microscopy (SEM, XL30ESEM-FEG, FEL, Netherlands). The size distribution of microspheres was determined by a SEM-scanning method reported earlier [18]. Briefly, the size distribution of microspheres was evaluated by a coefficient of variation (CV), which was calculated by following equation:

$$\mathrm{CV}=\frac{\mathrm{SD}}{\bar{D}}\times 100\%$$

The diameters of chitosan microspheres were evaluated by measuring a random vision field of 100 microspheres. Where $\bar{D}$ is the arithmetic average diameter of the total microspheres, SD stands for the standard deviation of the diameters.

Porosity values of the polymer scaffolds were measured by a modified liquid displacement method [19].

The porous scaffolds were cut into small rods of 15 mm × 2 mm × 1.5 mm. Each rod was bent to fit the incisor sockets, and sterilized with a 25 kGy Co60 radiation in preparation for *in vivo* experiment.

### *In vitro* release study

The ADM release profiles from microsphere-scaffold composite were carried out *in vitro* at 37°C in 10 mL of a modified stimulated body fluid (SBF) solution dynamically for 12 weeks. The SBF solution is similar to that recommended previously [20]. The microsphere-scaffold composite with ADM was immersed into the SBF. The release medium was withdrawn at each predetermined time interval, and replaced with fresh soaking medium (3 ml) each time. The elution of ADM in release medium was examined every a week by high performance liquid chromatography system (HPLC, Waters 2695, Milford, USA). The PLGA/nHA scaffold loading with ADM without chitosan microsperes was set as control. All of the data presented in the figure are the average data from 6 parallel samples.

### Animals and surgical procedures

Fifty-four male Wista rats (8 week old) with average weight 250–300 g were used. The rats were fed in Jilin University Laboratory Animal Center under similar experimental conditions. The animal protocol for this research was followed by Principles of laboratory animal care (NIH publication No. 85–23, revised 1985), and approved by the Animal Care and Utilization Committee of the School of Stomatology, Jilin University as well. Rats were randomly divided into 3 groups: (1) experimental group (PLGA/nHA/CMs/ADM scaffold implant after incisor extraction); (2) control group (PLGA/nHA/CMs scaffold implant after incisor extraction); (3) blank control group (untreated after incisor extraction).

The mandibular right incisor was cut at a gingival level by a small diamond bur at high speed every three days before extraction under ether anaesthesia. Three days after last cutting, the right incisor was extracted carefully without any rotation under deep anesthesia with pentobarbital (40 mg/kg, intramuscular). Afterwards, the PLGA/nHA/CMs/ADM and PLGA/nHA/CMs scaffolds were implanted into tooth sockets of experimental group and control group, respectively. The blank control group was untreated after tooth extraction. The gingiva was closed with sutures. Antibiotic prophylaxis included 30 mg of penicillin preoperatively and 30 mg a day for 2 postoperative days via recommendation of the veterinarian department.

### Radiological examination

The rats were sacrificed at 4, 8, 12 week post-operation under deep anaesthesia. All rats were perfused via the ascending aorta with saline followed by 4% paraformaldehyde in 0.1 M phosphate buffer (pH 7.4). Thereafter, the mandible was dissected out and fixed in the same fixative solution for 2 days at 4°C.

Soft X-ray images of the mandibles was taken by soft x-ray radiographic apparatus (Giotto Image-MD by IMS, Bologna, Italy). The digital imagine was got by scanning (Epson perfection 4490 photo, Seiko Epson Corp., Nagano, Japan). The distances between the infradentale point (apex of mandible ridge of incisor) and the highest point of the mesial alveolar bone at the lower first molar on both side were measured with Image-Pro Plus software (Media Cybernetics, Silver Springs, MD). The ratio of the heights on the left and right side was used to determine the residual ridge resorption on the preservative sites after tooth extraction.

In addition, the bone mineral density (BMD) of mandibular alveolar bone was initially detected by Dual-energy X-ray absorptiometry (DEXA) for small animals, and then calculated. The area of interest for BMD evaluation was from the anterior edge of alveolar ridge to the proximal surface of the first molar, since there is no tooth root in this area.

### Histological examination

The mandibles were harvested at each predetermined time point. The progress of perfusion and fixation of specimens was performed as described above. Thereafter, we used a 10% ethylenediaminetetraacetic acid (EDTA) solution in PBS for decalcification, pH 7.4, for a period of 6–8 week at 4°C, depending on degree of mineralization, with renewal of EDTA every week, before the specimens were dehydrated in a series of alcohols, transferred to xylene and embedded in paraffin. Serial transverse sections of tooth socket (4 μm thick) were prepared for evaluation of newly bone formation. The first one of every 5 consecutive sections was mounted on a piece of polylysine-coated slide, and then staining with haematoxylin-eosin.

To investigate the bone formation performance of the developed scaffolds at *in vivo* state, histomorphometric measurement was performed under a DM 4000 light microscopy (Leica, Germany) using an image analysis program (Leica Q VIN3 Software). The following parameters were measured: Dimensions of total defect area and newly formed bone. The percentage of values also were obtained in relation to the total defect area:

$$\mathrm{Newly}\;\mathrm{Formed}\;\mathrm{Bone}\;\left(\mathrm{NFB}\%\right)=\mathrm{New}\;\mathrm{bone}\;\mathrm{area}/\mathrm{Total}\;\mathrm{defect}\;\mathrm{area}\times 100\%$$

### Statistical analysis

All values were expressed the mean ± standard deviation (SD). Statistical analyses were carried out using SPSS 17.0 for Windows (SPSS Inc., Chicago, IL, USA). The statistical significance of differences was assessed by one-way analysis of variance. Statistically significant values were defined as P < 0.05.

## Results

### Characterization of chitosan microspheres and PLGA/nHA/CMs scaffolds

**Figure 2:**
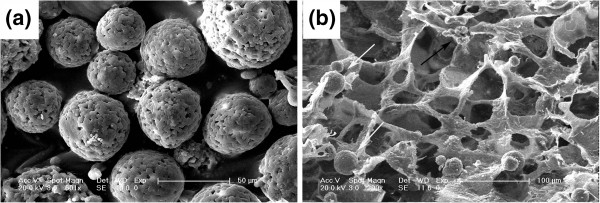
**SEM images of chitosan microspheres with ADM (a) and PLGA/nHA scaffold (b) prepared in presence of CMs-ADM.** The arrows show the chitosan microspheres (white) and nHA (black) in the scaffold.

**Figure 3:**
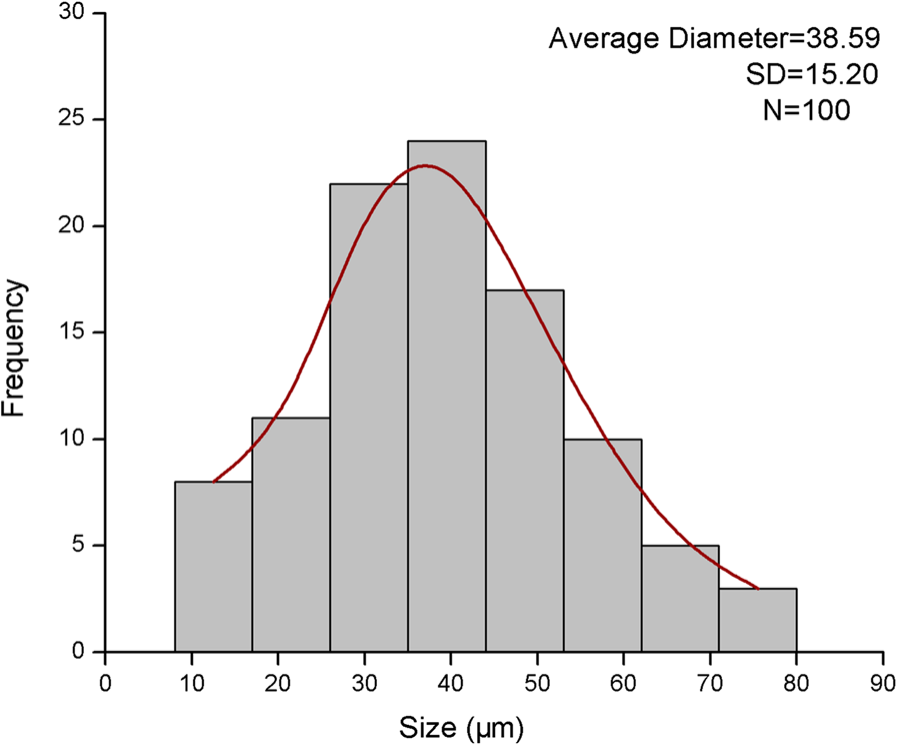
**The size distribution of chitosan microspheres with ADM loading.** The diameter of the microspheres was ranged from 8 μm to 75 μm, which confirmed to a normal distribution.

Chitosan microspheres were prepared in presence of TPP by ionic cross-linking between a carrying five-negative-charge TPP and a positively charged amino group of chitosan in anaqueous acidic solution (Figure 1b). The surface morphology of chitosan microspheres with ADM was shown in Figure 2a. The microspheres had preferably a spherical structure spherical in sharp and a relative rough and uniformity surface without any hollow and crack. The diameters of CMs were obtained by measuring a random vision field of 100 microspheres from SEM images. The CV was 0.394, indicating a uniform size distribution. Moreover, as shown in Figure 3 combining with Figure 2a, the size of most microspheres varied from 8 μm to 75 μm. The particle size distribution also confirmed to a normal distribution, with the average diameter of (38.59±15.20) μm.

The morphology of PLGA/nHA scaffolds with ADM-CMs incorporation was shown in Figure 2b. The SEM image showed the CMs were distributed all across the PLGA/nHA matrix. The scaffolds were seen to have open and interconnected pores. Furthermore, most of pore sizes of the scaffolds were located between 50 μm and 220 μm. In addition, the porosity of PLGA/nHA/CMs/ADM was (88.93±0.32) %.

### *In vitro* release kinetics of ADM

**Figure 4:**
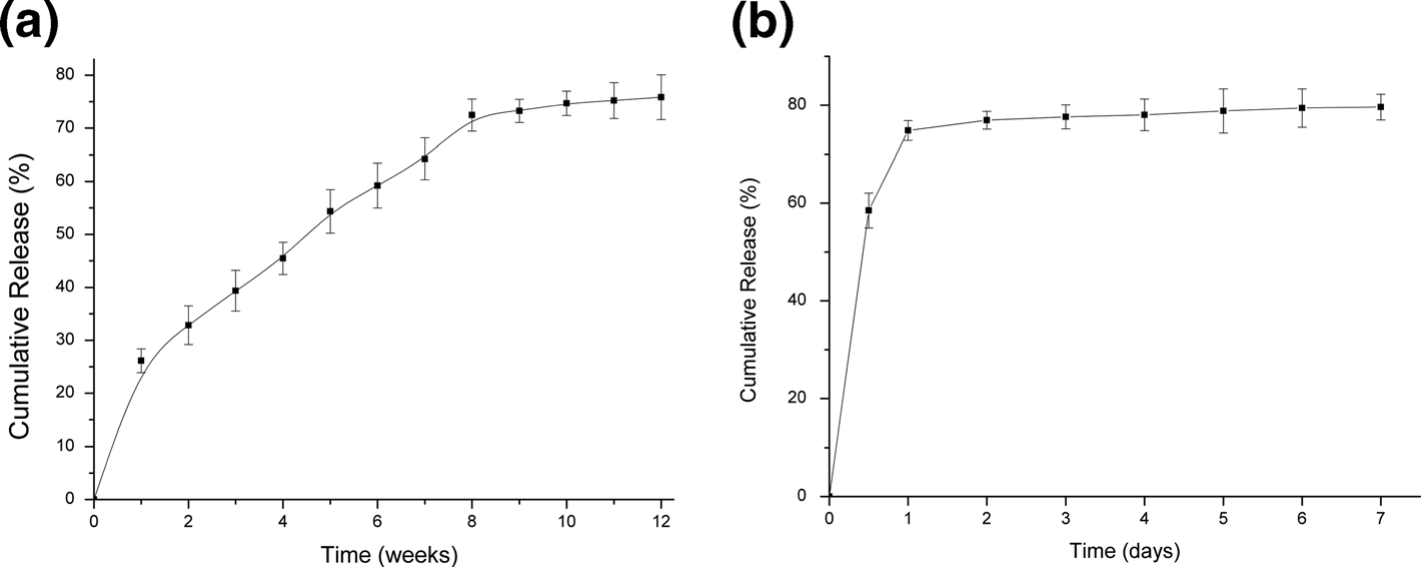
***In vitro *** **release of ADM from PLGA/nHA/CMs scaffold (a) and PLGA/nHA scaffold (b) in SBF solution.**

The release kinetics of ADM from PLGA/nHA/CMs and PLGA/nHA porous scaffold was depicted in Figure 4a and 4b. The release behavior of ADM from scaffold with chitosan microspheres tended to be a sustained linear manner during the first 8 week after an initial burst at 1 week, followed by a slow and stable manner from 8th week. As a result, the final amount of released ADM out of scaffolds was 68.94% within the whole stages. However, an initial burst of 74.8% was appeared at day 1 in the control group, followed by a plateau with a final cumulative release of 79.6% at day 7.

### Relative height of residual alveolar ridge

**Figure 5:**
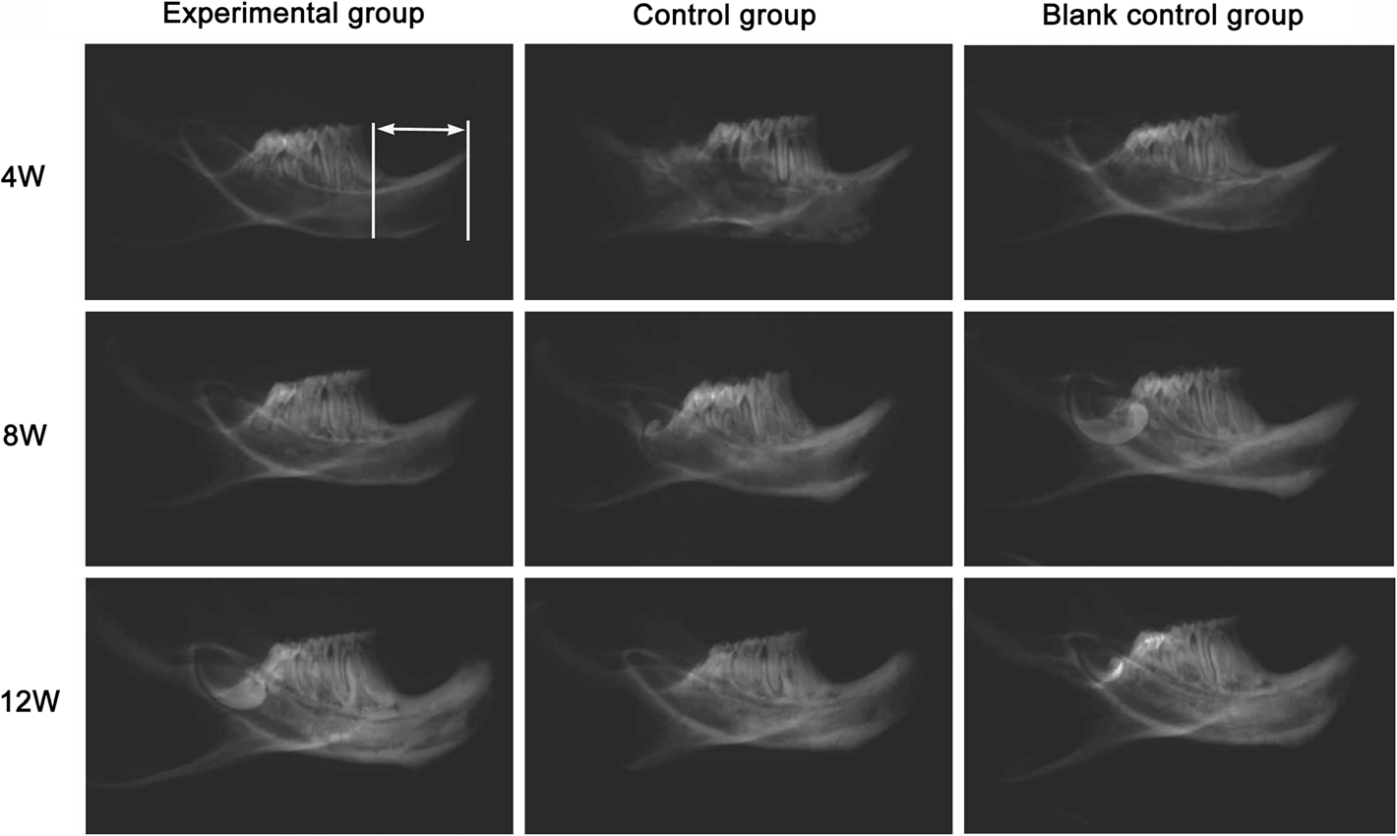
**Soft X-ray images of mandibles at each predetermined time.** The morphometric measurement on residual alveolar ridge by radiation is focused on the distance between apex of mandible ridge of incisor and the highest point of the mesial alveolar bone of first molar, just as the arrow showed.

The distance of interest was used to evaluate the relative height of residual alveolar ridge (Figure 5). Relative height values of residual alveolar ridge in all groups were shown in Table 1. Soft radiographs, shown in Figure 5, displayed the operative sides of mandibles at predetermined times. The values in the experimental group were significantly higher compared with those of the other two groups at 4, 8, 12 week post-operation. Meanwhile, the values of the control group were significantly higher compared with those of blank control group at 8, 12 week post-operation.

**Table 1:** The relative height of residual alveolar ridge after tooth extraction

**Time (weeks)**		**Group**	
	**Experimental group**	**Control group**	**Blank control group**
4	0.9890 ±0.0079^*#^	0.9651 ±0.0075	0.9612 ±0.0126
8	0.9828 ±0.0093^*#^	0.9495 ±0.0116^#^	0.9317 ±0.0103
12	0.9579 ±0.0101^*#^	0.9213 ±0.0166^#^	0.8992 ±0.0155

Values are expressed as means ± SD (n = 6). *p < 0.05, significantly different compared with control group, # p < 0.05, significantly different compared with blank control group.

### Bone density examination

The BMD values in different groups were presented in Table 2. Corresponding to the Soft X-ray images (Figure 5), BMD values appeared no difference among the three groups. At 8 week post-operation, the values were significantly higher in the experimental group and control group than blank control group. At 12 week post-operation, only BMD value in the experimental group was significantly higher than the other two groups.

**Table 2:** **BMD values of mandibular alveolar bone (mg/cm**^**2**^**)**

**Time (weeks)**		** Group**	
	**Experimental group**	**Control group**	**Blank control group**
4	62.80±3.80	60.97±5.28	59.89±2.25
8	81.72±3.22 *^#^	75.91±1.20^#^	69.83±1.98
12	93.10±1.80^*#^	88.08±1.23	86.33±1.31

Values are expressed as means ± SD (n = 6). *p < 0.05, significantly different compared with control group. # p < 0.05, significantly different compared with blank control group.

### Histological examination

**Figure 6:**
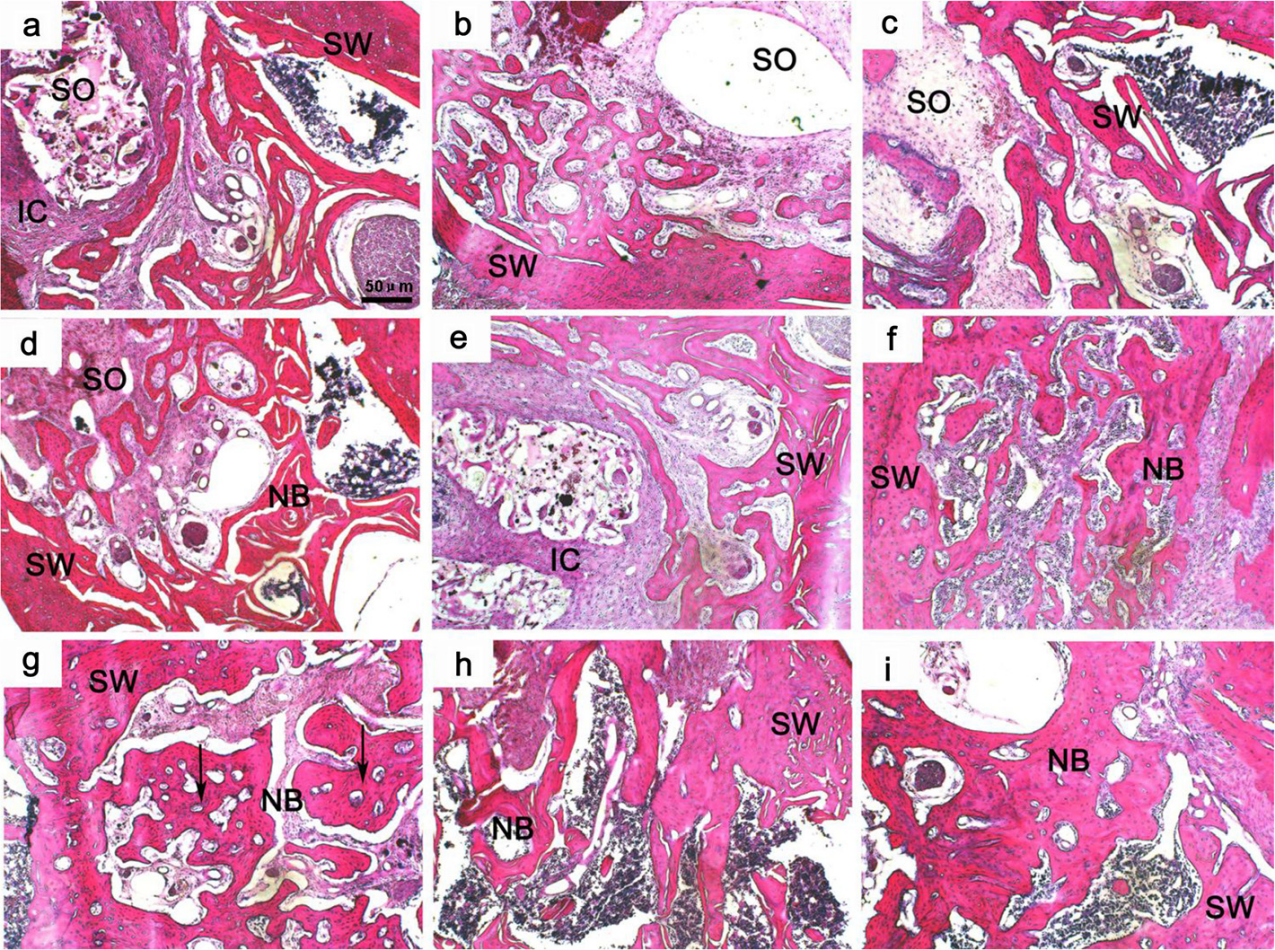
**Histological images by HE-stained tissue sections of tooth socket at different times after implanting operation.** (**a, d,** and** g**) the PLGA/nHA/CMs/ADM group (4, 8, and 12 weeks, respectively); (**b, e,** and **h**) the PLGA/nHA/CMs group (4, 8, and 12 weeks, respectively); (**c, f,** and **i**) the blank control group (4, 8, and 12 weeks, respectively); NB: newly formed bone, SW: socket wall, SO: tooth socket, IC: inflammatory cells filtration, Arrows indicate bone lacuna. The scale bar: 50 μm.

Histological evaluation was performed with HE staining of tooth extraction socket in mandible including the different implanted scaffolds, and the result was shown in Figure 6. At 4 week post-operation, mild inflammatory reaction was found in tooth socket in all groups. The tooth socket was fully filled with fibrous tissue. The scaffolds with a little tissue ingrowth were observed to be encapsulated by a connective tissue layer which was composed of fusiform stroma cells, macrophages, inflammatory cells and extracellular matrix. Only a layer of primary trabecular bone at the margin of the tooth socket was observed (Figure 6a, 6b, 6c). At 8 week post-operation, the PLGA/nHA/CMs with ADM was repalced by creeping substitution and a lot of fibrous callus appeared in the tooth extraction socket. More newly formed bone which tended to penetrate into the scaffold was found, and the scaffold was degraded into fragmental pieces as well (Figure 6d). In contrast, less primary bone was observed in the boundary area between the scaffold and alveolar bone in the control group. The scaffold surrounding with a dense cell layer was still occupied the defect regions (Figure 6e). Furthermore, although there exsited inflammatory cell infiltration, the newly formed bone was more mature in blank control group (Figure 6f). At 12 week post-operation, the scaffold appeared an obvious degradation accompanied with the new bone formation. Especially in experimental group, bone islands, which were fused to the original bone around the defect, formed in the defect areas. In addition, bone lacunae, which is a mark of the new bone formation contained osteocyte-like cells, were also appeared. The texture of bone trabecula was remarkably clearer than that in thw control group (Figure 6g). On the contrary, a large amount of original osseous callus formed in the tooth socket, and yet the space with scaffold occupied also exsited in the control group (Figure 6h). Moreover, in the blank control group, the inflammory cell infiltration was disappeared, while massive mature newly formed bone grown into the center of the tooth socket. However, there were quite a few spaces without any osteoblastic activity (Figure 6i).

**Figure 7:**
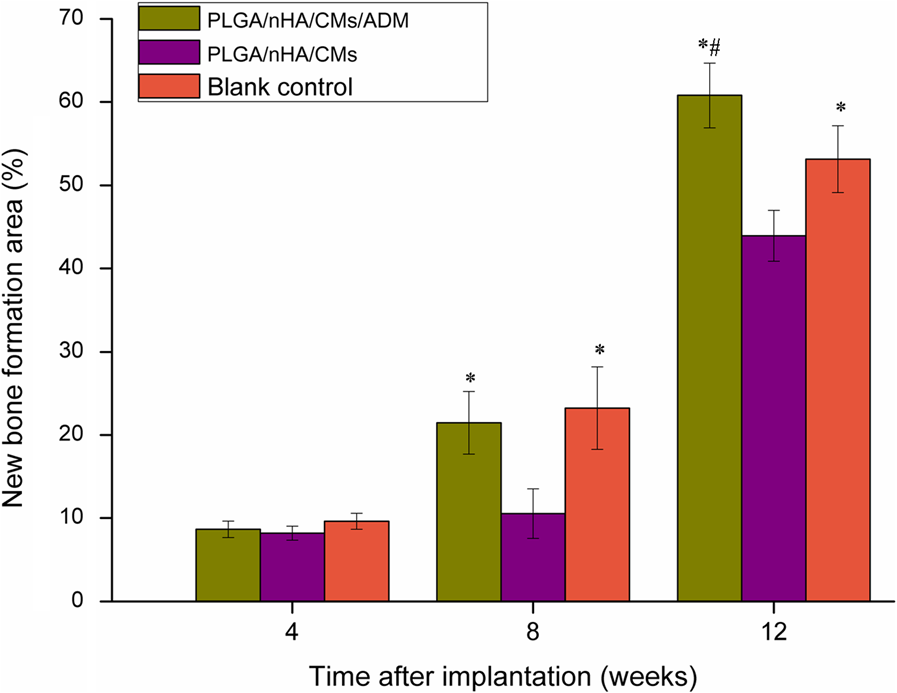
**Bone formation areas of various scaffolds at 4, 8 and 12 weeks after implantation.** Mean values were compared by ANOVA and were significantly different at P<0.05. *statistically significant different compared with PLGA/nHA/CMs group. #statistically significant different compared with blank control group.

In order to quantity the bone formation performance of the scaffolds at *in vivo* state, histomorphometric test was carried out. The results were shown in Figure 7. In terms of osteoid formation, the statistically significant differences were appeared in PLGA/nHA/CMs/ADM group and blank control group compared with PLGA/nHA/CMs group at week 8 and 12. Meanwhile, the value of the experimental group was significantly higher than that of blank control group at week 12 post-operation.

## Discussion

Due to the adrenomedullin’s short clearance time *in vivo*, a controlled delivery system was developed to load it. Although there was a report that PLGA scaffold solely could be used for protein controlled delivery [21], PLGA/nHA scaffold loading with ADM leads to an initial burst of 74.8% during the first day in our study. Therefore, chitosan microsphere was introduced into PLGA/nHA scaffold to be a newly microsphere-scaffold delivery system. Chitosan microspheres loading with ADM were prepared by an emulsion-ionic cross-linking method in presence of TPP. Unlike chemical cross-linking, this method can help to to avoid negative effects for proteins and peptides. Cross-linking time is critical to formation of microspheres, because the combination of TPP molecules with microspheres is time-dependent. If the cross-linking time is not long enough, only fragile microspheres with irregular surface will be obtained. Microspheres under a normal distributed size with an average of 38.59 μm might be suitable for further usage in PLGA/nHA scaffold. PLGA as a biodegradable material has been used as bone regenerative materials in surgery for a long time. An ideal porosity of more than 80% is a distinct symbol of a perfect scaffold [22]. The high porosity of PLGA/nHA/CMs/ADM was perhaps a consequence of an interconnected three-dimensional pore structure. A pivotal study by ELEMA *et al.*[23] showed that optimal pore size for bone ingrowth was in the range of 75–250 μm, since too small a size would obstruct diffusion of essential nutrients and oxygen for cell survivability and too large a size would decrease the local medicine concentration. In this study, open and interconnected pores with size ranged from 50 μm to 220 μm were established by thermally induced phase separation, which provided a large surface area to allow cell attachment and facilitated nutrient and waste exchange by cells deep within the construct. Furthermore, the pore size within the optimal range was suitable for cell penetration.

The release behavior of ADM from scaffold with chitosan microspheres tended to be a sustained linear manner during the first 8 week, followed by a slow and stable manner to 12th week. The plasma half-life of ADM has been reported to be 22.0±1.6 min [24], and corresponding to the release profile and total amout of ADM, the released ADM concentration would maintain at 1 nM, which could be optimal to osteoblast proliferation *in vitro*. The initial burst release in the first week may due to the physical absorption of ADM on the scaffold surface. This microsphere-scaffold delivery carrier could maximize the effect of ADM by maintaining the local peptide concentration at a designed level for a longer period according to continuous and sustained release. The PLGA/nHA/CMs microsphere-scaffold system has several superiorities as follows: Firstly, the nHA played a role in mimicking the nanostructure of natural bone. Secondly, alkaline dissolution of the chitosan microsphere could neutralize the degraded acidic molecules of PLGA, which might decrease the negative effect of PLGA degradation. Moreover, the progress of making microsphere-scaffold carrier is non-toxic, which could preserve the bioactivity of the introduced peptides and proteins. Consequently, it could be predicted that the local delivery of ADM by microsphere-scaffold carriers would be potent for further *in vivo* studies.

Bone formation process is a complicated cascade that relies on several mechanisms including synthesis of matrix proteins and calcium phosphate. The synthesis happens in a continuously renewed biological environment and is regulated by a cluster of growth factors [25]. ADM has been shown to induce angiogenesis *in vitro* and *in vivo*, which is essential to bone formation in tissue engineering [26,27]. However, the effect of ADM on bone resorption is controversial: GONZALEZ *et al.* documented that ADM treatment could inhibit the osteoclast-mediated activity [28], whereas others failed to observe any reduction in bone resorption when ADM was administered either locally or systemically [29,30]. To be sure, ADM is a potent osteoblast mitogen at a physiological concentration of 1 pM or greater.

The height and width of the residual alveolar ridge would decrease following tooth extraction. The morphometric analyses demonstrated that the relative height of the experimental group was significantly higher than that of the other groups at 4, 8, 12 week, which inferred that ADM could have effects on reducing the residual ridge resorption at the early stage. Also, the values of control group were significantly higher than those in blank control group at 8, 12 week. Perhaps, the PLGA/nHA/CMs can also maintain the relative height of residual ridge by its excellent osteoconductivity. Its effect on resorption prevention, however, is clearly weaker than the scaffold with ADM. It is logical to presume that ADM may contribute to the preservation of alveolar bone at tooth extraction site.

Bone mineral density (BMD) is an important index for assessing the amount of bone formation, as well as the degree of calcification of newly formed bone. Dual energy X-ray absorptiometry (DEXA) is currently the most widely used technique for bone loss measurement, and even has been an established standard for measuring bone mineral density. In the present study, DEXA analyses showed that the BMD of alveolar bone in experimental group was no significantly difference compared with the other two groups at 4 week, following with a distinct increasing at 8 ,12 week, which was significantly higher than the other groups. Apparently, functional occlusion is crucial for preserving the volume and structure of the mandible. After the incisor extraction, the BMD in blank control group would decrease conspicuously, whereas the presence of an osteoconductive bioactive scaffold can support the contour of the socket and allow osteoblasts to migrate to form bone more efficiently within the extraction space, which facilitates bone healing [31]. Interestingly, BMD was also higer in the experimental group at 12 week, corresponding to the ADM release profile, indicating that even if the releasing of ADM almost stopped, the higher BMD was still maintained, possibly because of the different release behavior between *in vivo* and *in vitro*. This different release behavior *in vivo* compared to *in vitro* was due to the enzymes, liposomes, germs and phagocytes *in vivo*.

To get a further insight, we were aimed to study the histological features of the tooth extraction socket. The physiological healing of tooth extraction socket is a complicated dynamic progress, beginning with a cascade of inflammatory reactions activated immediately after tooth extraction and ending with fully muture hard tissue formed in the tooth socket. Just as shown in blank control group, it occurs as five different stages, including the coagulum of blood cells, orgnization of blood clot and formation of granulation tissue, connective tissue replacement, start of calcification and mineral tissue deposition. Evidence has accumulated that most artificial scaffolds were effective for ridge preservation [32,33]. In the present study, there was no statistically significant difference among the three groups at 4 week post-operation according to the histomorphometric analysis, which was consistent with the BMD measurement. The graft material had no obvious degradation in the first 4 weeks. 8 weeks post-operation, significant differences have been observed in PLGA/nHA/CMs/ADM group and blank control group compared with the PLGA/nHA/CMs group. More mineral tissues and less inflammatory reactions were founed in PLGA/nHA/CMs/ADM group, while few primary bone was found in PLGA/nHA/CMs group. It inferred that PLGA/nHA/CMs scaffold had good osteoconductivity, but might lack of osteoinductivity in its current format. The ADM incorporation might enhance the osteoinductivity of the scaffold. ADM has been reported to function on not only inducing the formation of granulation but also improving local circulation, with the result of having angiogenesis accelerated [34,35], which is crucial in the bone healing process. The similar tendency of new bone histomorphometric analysis was appeared at 12 weeks after operation. However, the new bone formation area of the PLGA/nHA/CMs/ADM group was significantly higher than that of blank control group. Bone islands were discovered in the PLGA/nHA/CMs/ADM group and blank control group which revealed that ADM could accelerate bone mineralization and osseous maturation. This result was possibly due to the microsphere-scaffold system offered a suitable micro-environment for cell growth and tissue formation. It is hypothesized that the biomechanical similarity between PLGA scaffolds and human cancellous bone, could have contributed to *in vivo* osteogenesis, by providing a suitable environment for stem cells to differentiate into the osteogenic lineage. On the whole, the osteogenic effect in the experimental group was quick in speed and large in amount. As a result, the microsphere-incoporated-into-scaffold design offered a new strategy for local application of growth factors.

## Conclusions

In conclusion, this study demonstrated the microsphere-scaffold composite is a satisfactory delivery system for the sustained release of peptides. In addition, local application of adrenomedullin by microsphere-scaffold composite could enhance the formation of new bone in the tooth socket and maintain the residual alveolar ridge height in rats. The results may relate to the osseogenic and angiogenic properties of adrenomedullin. ADM may be a potential treatment for prevention of residual alveolar ridge resorption. Future studies will focus on specifying the signaling for osteoinduction of ADM. Thus controlled release profiles can be spatio-temporally designed to coordinate with signal transduction *in vivo*.

## Abbreviations

ADM: Adrenomedullin; PLGA: Poly (lactic-co-glycolic) acid; nHA: Nano-hydroxyapatite; CMs: Chitosan microspheres; TPP: Tri-polyphosphate sodium; PBS: Phosphate buffered saline; SBF: Stimulated body fluid; HPLC: High performance liquid chromatography; SEM: Scanning electron microscopy; BMD: Bone mineral density; EDTA: Ethylenediamine-tetraacetic acid; SD: Standard deviation.

## Competing interests

No conflict of interests is present. The authors have no financial involvement or interest with any organization or company about subjects or materials discussed in the paper.

## Authors’ contributions

LW carried out the synthsis and characterization of the scaffold-microsphere composite. LZ carried out the animal exiperiment including the animal model establishment, sample implant and specimen harvest. CL conceived of the study, and carried out the release behavior of the composite. SD participated in the design of the study and performed the statistical analysis. LA conceived of the study, and participated in its design and coordination and helped to draft the manuscript. YZ made substantial contributions to conception and design of this study and gave final approval of the version to be published. All authors read and approved the final manuscript.
